# Development of magnetic bead based sample extraction coupled polymerase spiral reaction for rapid on-site detection of Chikungunya virus

**DOI:** 10.1038/s41598-020-68469-2

**Published:** 2020-07-15

**Authors:** Shashi Sharma, Deepak Pardasani, Paban Kumar Dash, Manmohan Parida, Devendra Kumar Dubey

**Affiliations:** 1 Virology Division, Defence Research Development Establishment, Jhansi Road, Gwalior, 474002 India; 2 Vertox Division, Defence Research Development Establishment, Jhansi Road, Gwalior, 474002 India

**Keywords:** Microbiology, Molecular biology

## Abstract

The molecular detection system has evolved over last two decades and is rapidly replacing the conventional confirmatory techniques in diagnostic virology. However the major limitation in implementation of available molecular detection assays is the non availability of field deployable nucleic acid isolation platform coupled with gene amplification technique. The rapid and early molecular detection is crucial for employing effective measure against many viral infections. The re-emergence of chikungunya virus (CHIKV) has led to epidemics since 2004 in several parts of the world including India. The main association of CHIKV with severe arthritis and long-lasting arthralgia and closely mimics symptoms of Dengue and Zika virus infection requiring laboratory confirmation. In this study, a simple magnetic bead based ribonucleic acid extraction method was optimized, which was coupled with isothermal polymerase spiral reaction (PSR) technique for early and rapid detection. Subsequently, the polymerase spiral reaction reagents were converted to dry down format that led to a rapid user friendly field compatible sample processing to answer method for rapid and onsite detection of Chikungunya virus. Both the methods were evaluated with a panel of clinical samples. The sensitivity of the assays were compared with available commercial viral RNA extraction platform and qRT-PCR. The in-house nucleic acid extraction system based on magnetic bead followed by dry down RT-Polymerase Spiral Reaction assay was found to be highly sensitive with 10 copies of RNA as limit of detection in CHIKV clinical specimens. With respect to other closely related viruses no cross reactivity was observed. This novel methodology has the potential to revolutionize the diagnosis of infectious agents in resource limited settings around the world.

## Introduction

Molecular diagnostics based on nucleic acid amplification technology (NAT) has revolutionized the disease diagnosis over last three decades. Evolution in nucleic acid amplification technology since the invention of polymerase chain reaction (PCR) has revolutionized the world^1^. However, PCR including nested PCR and real-time PCR has their own limitation of the cycling for heating and cooling with a need of highly précised equipments. Nucleic acid extraction is another major bottleneck in the development and inclusion of all molecular diagnostic systems. Currently molecular diagnostics are restricted to few advanced laboratory owing to these limitations^2^.

Although the development of Real-Time Polymerase Spiral Reaction has led to development of rapid detection platform, yet performing manual isolation of nucleic acid makes it a very labor intensive excersise^3^. Phenol–chloroform extraction and ethanol precipitation methods are time-consuming, complicated, hazardous, and not suitable for processing of large numbers of samples. These traditional methods are widely adopted with the supplement of extraction procedure based on principle of adsorption of nucleic acids to silica matrices in presence of chaotropic salts and alcohol^4^. In this study, silica coated magnetic bead and formulations of alkaline, acidic and neutral buffers for binding and elution of ribonucleic acid from clinical samples was attempted. This method offers a rapid and field compatible nucleic acid extraction employing in-house ingredients, obviating the requirements of any high end equipments.

Isothermal gene amplification techniques are increasingly becoming popular in resource limited settings due to its simple and isothermal operation^5^. In the recent years various isothermal amplification techniques have been reported for amplification of nucleic acid including both DNA as well as RNA. However, only a few of these isothermal amplification methods such as Loop-mediated Isothermal Amplification (LAMP) was widely adopted and very efficiently performed at a constant temperature using a single enzyme but suffers from the requirement of four or more primers. The limitations of other isothermal assays viz., nucleic acid sequence-based amplification (NASBA) and helicase-dependent amplification (HDA) includes requirement of multiple enzymes to initiate a reaction^6–8^.

A novel isothermal method Polymerase spiral reaction (PSR) uses two primers and a DNA polymerase enzyme for amplification. Since the reaction is generally performed at a single temperature, therefore need of an energy intensive thermal cycler can be minimized^9^. The result interpretations can similarly be performed with changes in color visually. The applicability of the PSR assay has been shown for an on-site as well as point of care testing in human and veterinary health care settings^10–13^.

Emergence and re-emergence of chikungunya virus (CHIKV) as an important pathogen has caused a large scale epidemics of acute febrile arthritis in both urban and rural settings across many parts of the globe, including Europe, Americas, Africa and Asia^14–16^. CHIKV, a member of genus *Alphavirus* belonging to family *Togaviridae* is the etiology of Chikungunya infection. The Chikungunya virus is an enveloped, positive-strand RNA virus of approximately 12,000 nucleotides^6^. The disease typical clinical sign includes poly-arthralgia which is very painful. Severe complications including neurological manifestations were also reported from several cases. A small proportion of patients may report arthralgia persisting for several months^17^. Rapid molecular diagnosis is therefore warranted for better patient management and avoids severe complication of the disease^18^.

Several reports are available of rapid and real time detection of Chikungunya virus in clinical samples^19–21^. Loop-mediated isothermal amplification assay (LAMP) is a widely adopted isothermal assay has been reported^22^, however the adaptability of these fast nucleic acid detection platforms is limited due to absence of simple and reliable sample extraction method. It therefore seeks attention towards development of a rapid, user friendly, on-site sample to answer detection platform. Here we report the development of an in-house assay comprising of magnetic bead based RNA extraction and lyophilized RT-PSR reagents for rapid onsite detection for Chikungunya. This technology was also compared with a widely used commercial RNA extraction kit coupled with laboratory based SYBR Green qRT-PCR platform to evaluate its clinical utility.

## Results

### Extraction of viral RNA employing silica coated magnetic bead

Initial experiments were performed with silica coated magnetic bead nucleic acid extraction protocol along with commercially available QIAamp viral RNA kit in order to study quality and quantity of RNA. The whole set up of in-house magnetic bead based nucleic acid extraction protocol was represented in Supplementary Fig. S1. Duration of whole procedure was 30 min for viral RNA extraction without use of any electrical equipment. The comparative evaluation revealed the concentration of RNA to be 20 ng/µl for in house magnetic bead protocol, whereas it was 18 ng/µl in Qiagen protocol. Quality of RNA as determined through A_260/280_ was 2.4 and 3.2 in magnetic bead and Qiagen protocol respectively. The suitability of extracted RNA from both protocols for molecular assay (SYBR Green qRT-PCR) was also confirmed, as demonstrated by positive reaction through amplification curve analysis ABI7500Dx real time RT-PCR system. The nucleic acid extracted from a panel of samples through both these methods revealed 100% concordance which was confirmed though CHIKV E1 specific SYBR green qRT-PCR. The Ct value obtained is summarized in Table 1.

**Table 1:** Comparison of viral RNA extraction protocol using silica coated magnetic bead and Qiagen viral RNA kit from Chikungunya isolate and positive clinical samples.

S no.	Details	Magnetic bead extraction + SYBR Green qRT-PCR (Ct value)	Qiagen extraction + SYBR Green qRT-PCR (Ct value)
1	RNA extracted from CHIKV isolate	23	23
2	RNA extracted from CHIKV positive serum sample	26	25.5
3	RNA extracted from CHIKV negative serum sample	No Ct	No Ct
4	PTC	22	22
5	NTC	No Ct	No Ct

### Reverse transcription-polymerase spiral reaction (RT-PSR)

Primers were designed targeting the E1 gene and the same was found conserved in all CHIKV genotypes. Cross reactivity of primer sets were also ascertained. Standardization and optimization of the RT-PSR assay for various temperature and time was carried out. The optimum results were observed for CHIKV specific RT-PSR at 65 °C for 60 min both in digital heat block (Supplementary Fig. S2A) and real time turbidimeter (Supplementary Fig. S2B). Positive reaction was observed by bright green fluorescence on addition of SYBR Green I dye, while the negative sample retained the original orange color (Supplementary Fig. S2C). Under UV light, the color was intensified as bright green fluorescence in positive control while, no fluorescence was observed in negative control (Supplementary Fig. S2D). Amplification curve was found in PTC, whereas no such curve was visible in NTC, when the reaction was performed using Loopamp real time turbidimeter (Supplementary Fig. S2E). Distinct laddering pattern was visualized in the positive samples when they were resolved in 2.5% agarose gel (Supplementary Fig. S2F). The reagents were subsequently lyophilized using 50% D-Trehalose (Supplementary Fig. S3A). The PTC revealed green fluorescence in both naked eye and under UV light, whereas NTC retained the orange color (Supplementary Fig. S3B,C). The same was also confirmed through appearance amplification curve in PTC and its absence in NTC (Supplementary Fig. S3D). Distinct laddering pattern similar to wet reagents was visualized in 2.5% agarose gel (Supplementary Fig. S3E). The lyophilized RT-PSR reagents were tested with PTC, NTC and clinical samples (Supplementary Fig. S3F). The replicates of batches prepared with 50% trehalose were found stable at 4 °C up to 6 months as revealed through change of color (Supplementary Fig. S4A-C1-3). However, the lyophilized RT-PSR reagents were found stable only up to 1 week, when stored at room temperature (Supplementary Fig. S4C4).

**Figure 1:**
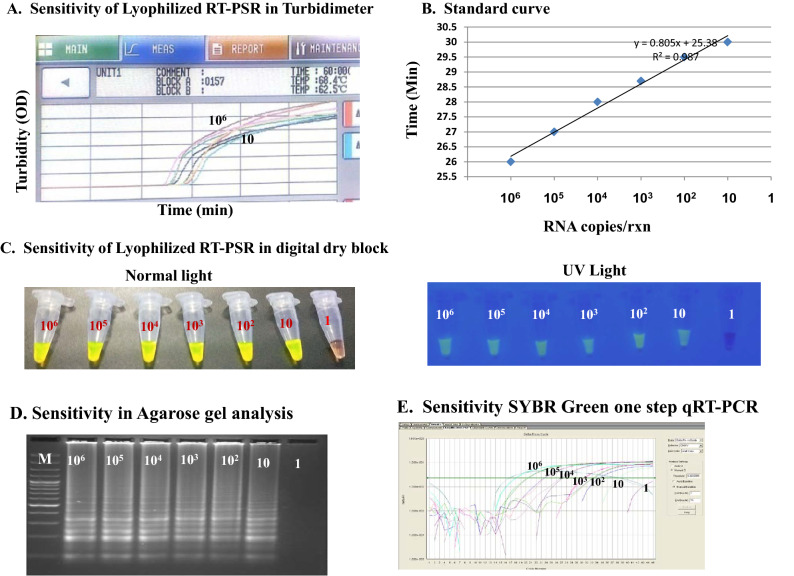
Sensitivity of lyophilized RT-PSR and SYBR green qRT-PCR using tenfold serial dilution of CHIKV targeted in vitro transcribed RNA. (**A**) Sensitivity of lyophilized RT-PSR in real time turbidimeter indicating a LOD of 10 RNA copies/rxn. (**B**) Standard curve obtained by plotting the value of time of positivity in real time turbidimeter versus RNA copies/rxn. (**C**) Sensitivity of lyophilized RT-PSR in digital dry bath indicating a LOD of 10 RNA copies/rxn. Visual detection through naked eye in normal light and UV light. (**D**) Sensitivity of lyophilized RT-PSR in 2.5% agarose gel indicating a LOD of 10 RNA copies/rxn. (**E**) Sensitivity of SYBR Green one step real time qRT-PCR indicating a LOD of 10 RNA copies/rxn.

The detection limit of lyophilized RT-PSR was found to be 10 RNA copies/rxn (equivalent to 714 RNA copies/ml of sample), when performed in both tubidimeter and digital dry block (Fig. 1A–C). The typical laddering pattern was observed on gel electrophoresis (Fig. 1D). When the same dilutions of IVT RNA were subjected to SYBR green real time qRT-PCR, the detection limit was also found to be 10 RNA copies/rxn (Fig. 1E). RT-PSR assay didn't show any amplification with closely related alphaviruses (O’nyong-nyong virus, Semliki Forest virus, Ross River virus, and Sindbis Virus) and flaviviruses (Dengue virus serotypes 1–4, Japanese encephalitis virus, and West Nile virus), included in this study (Supplementary Fig. S5). The templates of alphaviruses were confirmed through presence of 434 bp amplicon through end point RT-PCR; whereas flaviviruses were confirmed through virus specific real-time RT-PCR (Supplementary Figs. S6 and S7).

### Comparison between magnetic bead based RNA extraction coupled RT-PSR and Qiagen RNA extraction coupled SYBR Green real time RT-PCR assay

**Figure 2:**
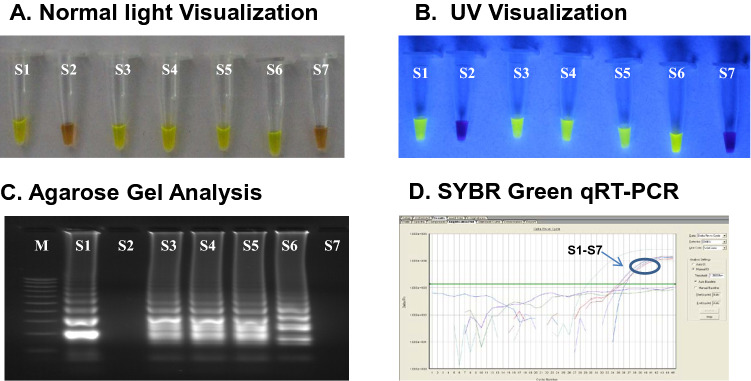
Analysis of clinical samples by Lyophilized RT-PSR and SYBR green qRT-PCR. (**A**) Visualization through naked eyes, following addition of SYBR green. (**B**)Visualization in UV light, following addition of SYBR green. (**C**) Agarose Gel analysis. (**D**) SYBR Green qRT-PCR.

The dry down reverse transcription-polymerase spiral reaction method was evaluated simultaneously SYBR Green one step real time RT-PCR using a panel of 70 suspected CHIKV serum samples. The result revealed 94.25% concordance between both the methods. Out of 50 positive samples, 46 were found positive by both the methods. However 4 samples of low viral load were missed by dry down RT-PSR assay, indicating a sensitivity of 92%. However, none of the negative and healthy sample showed any signal of positivity, indicating a specificity of 100% (Table 2, supplementary Table S1). Analysis of seven representative sample panel in lyophilized RT-PSR interpreted in normal light, UV light and 2.5% agarose gel was depicted in Fig. 2A–C. Same samples were also found positive in SYBR green qRT-PCR (Fig. 2D). Randomly selected samples (n = 10) found to be positive for both RT-PSR and SYBR Green real time PCR were subjected to DNA sequencing by Sanger’s method. BLAST analysis revealed the presence of CHIKV E1 genomic region in the specific products (Data not shown). Sequencing results thus confirmed the accuracy of PSR in detecting CHIKV in clinical samples.

**Table 2:** Comparative evaluation of Magnetic bead RNA extraction coupled lyophilized RT-PSR with Qiagen RNA extraction coupled SYBR green RT-PCR.

Magnetic bead based RNA extraction coupled lyophilized RT-PSR	Qiagen RNA extraction coupled SYBR green RT-PCR		
	Positive	Negative	Total
Positive	46	0	46
Negative	4	20	24
Total	50	20	70

Concordance = 94.25%, Sensitivity = 92%, Specificity = 100%.

## Discussion

Chikungunya is a serious international public health important infection. Availability of very few diagnostic kits limits its early detection in rural settings, where the disease is endemic^23,24^. The rapid and field compatible sample extraction is a bottleneck in development of rapid molecular detection systems. Another important concern is storage and transportation of these thermo-labile molecular reagents. Both these issues increase the cost and prohibit adaptation of rapid detection platforms in resource limited settings. Development of simple sample extraction along with detection employing lyophilized molecular reagents will not only help the transportation issues of temperature sensitive reagents but also aid in providing sample to answer. In this study, we report a unique aqueous chemistry to capture and purify nucleic acids on surface of magnetic bead. Clinical sensitivity and detection limits were compared with Qiagen viral RNA kit. Results revealed similar sensitivity with widely used commercial Qiagen kit. Further, this bead based extraction protocol can easily be implemented in field condition for onsite sample preparation without requiring any specific laboratory equipments.

At present, number of laboratory based diagnostic techniques is used to detect Chikungunya virus infection. This includes conventional culture based identification, immunological detection through direct ELISA and molecular detection by end point RT-PCR, qRT-PCR and RT-LAMP^19–22,25,26^. The conventional culture method is tedious and time consuming and also need expertise with well established cell culture facilities. Though serological techniques are frequently used but still suffer from false positivity due to circulation of cross reactive viruses. Therefore detection based on molecular assay is widely preferred throughout the world for confirmatory diagnosis. Polymerase spiral reaction is a recently reported field compatible onsite isothermal rapid molecular detection platform. PSR is a novel isothermal method that fulfils the requirements of World Health Organization (WHO) guidelines for development of diagnostic systems. The current objectives of developing diagnostic by research laboratories mainly lies in “ASSURED” (affordable, sensitive, specific, user friendly, robust and rapid, equipment free and deliverable) philosophy^9^. The aim is now shifting towards development of onsite point of care diagnostics. The main reason behind this is the revolution in molecular biology detection platforms including various isothermal diagnostic assays. PSR method has advantages over traditional PCR methods in comparison to time, cost, specificity, sensitivity; omit the gel electrophoresis, need for high end equipment and onsite detection capabilities. Specific nucleic acids can be amplified and detected in isothermal conditions, without need of any costly equipment. A positive reaction results in accumulation of large amount of pyrophosphate so that turbidity can also be recorded for quantification purpose. Primer designing for RT-PSR is very simple and similar to conventional end point PCR (i.e. identification of a conserved gene revealed through multiple sequence alignment). Further, sequence from an unrelated species that is an exogenous or from a botanical origin is appended at the 5′ end of the primer sequence at the target region. In contrast, LAMP, another isothermal technique requires complicated primer designing tool. Therefore the PSR method has the potential to be setup in rural settings as per requirements.

In this study, we coupled both a simplified magnetic bead based RNA extraction with RT-PSR for early detection of Chikungunya virus. The limit of detection of this dry down RT-PSR format was found to be 10 RNA copies/reaction, which is comparable to an off-site detection format i.e. real time SYBR Green qRT-PCR. The specificity and sensitivity of this developed assay was also found comparable to PSR assays earlier reported for other microbial agents^9–13^.

This assay reagents were further lyophilized in order to maintain reagent stability. We have optimized the reagent stability along with enzymes at 4 °C with addition of 50% trehalose as the stabilizer in reaction mix before lyophilization. We have observed that the lyophilized RT-PSR assay was stable at least up to 6 months at 4 °C and 1 week on room temperature. The fast and field based diagnostics including nucleic acid purification coupled rapid detection is an urgent demand in the present scenario for diagnosis of infectious diseases. A simplified aqueous chemistry for RNA extraction reported here will serve as a base method for integration of various available qRT-PCR and isothermal molecular detection platforms. A magnetic bead based simple and easy sample extraction method in conjunction with lyophilized RT-PSR reported here has enabled a rapid and field compatible molecular detection platform for Chikungunya virus. The novelty of this method lies in its simplified procedures and easier applicability in both laboratory as well field conditions. To the best of our knowledge, this is the first report of RT-PSR assay against a human RNA virus. The present work can be further extended to develop rapid user friendly sample to answer detection platform for other RNA viruses.

## Materials and methods

### Ethical statement

This study was performed in accordance with appropriate guidelines. The experimental protocols conducted in this study were approved by Defence Research and Development Establishment- Institutional Biosafety Committee, (DRDE-IBSC) vide no. IBSC/VIRO-06/05/PKD. All the experiments were carried out as per National Biosafety guidelines in a BSL-2 laboratory using appropriate personal protective equipments. The clinical samples were referred to DRDE from Gajra Raja Medical College (GRMC), Gwalior for laboratory diagnosis. Informed consent from suspected Chikungunya patients have been obtained, prior to collection of samples.

### Clinical samples

The serum samples from patients (n = 50) with suspected CHIK infection during outbreaks in different parts of India from 2015–2018 were referred for molecular diagnosis to DRDE, Gwalior from Gajra Raja Medical College (GRMC), Gwalior. The serum samples collected between day 1 to 7 after onsets of specific symptoms were used in this study. Along with this, apparently healthy serum samples were collected from healthy individuals (n = 20) were kept as negative control.

### Virus

Chikungunya virus (CHIKV DRDE-07, an Indian isolate, maintained at DRDE, Gwalior, India) was used as positive control in this study. Ross river virus (RRV), O’nyong-nyong virus (Gulu strain, GenBank Acc No M20303), Semliki Forest Virus (L10 strain, GenBank Acc No AY112987) and Sindbis virus (GenBank Acc No J02363) were included as representative member of Alphavirus in this study for assay specificity. Another representative members belong to Flavivirus group i.e.; the Dengue virus serotypes (DENV-1, RR107 (KF289072), DENV-2, GWL18 (AY324614), DENV-3, ND143 (FJ644564), DENV-4, ND 73 (HM237348)), Japanese encephalitis virus (JaOArS982), West Nile virus (Eg 101) co-circulating with similar clinical symptoms were also included in this study for evaluating the specificity of the assay.

### Magnetic bead particles

Magnetic bead particles were prepared using Stober’s method includes single step functionalization^27^. Five hundred milligram of Fe_3_O_4_ nanoparticles (Aldrich, USA, Cat. No. 544884) were sonicated (30 min) in a solution having 320 ml of ethanol and 80 ml of water followed by addition of 10 ml of 30% NH_4_OH solution. This solution was mixed with 2 g of tetraethylorthosilicate (Aldrich, USA, Cat No. 86578) for 45 min and the contents were allowed to stir at room temperature for additional 24 h. This silica coated nanoparticles were than separated under magnetic field, washing was done with water and ethanol and finally dried under vacuum (70 °C, 24 h). The average size of particles was found 2.3 µm and BET surface area was kept 17 m^2^/g. Finally these particles were used in a suspension of 50 mg of particles/ml in nuclease free water.

### Buffers

Three buffer solutions were employed for extraction of viral RNA viz*,* lysis/binding buffer (3.96% Ammonium sulphate, 0.8% NP-40 in 0.2 M Tris acetate, pH 4), washing buffer (0.5% NP-40 in 0.01 M Tris–HCl, (pH 6.8)) and elution buffer (10 mM Tris–HCl, (pH 8.5)).

### Extraction of viral RNA employing silica coated magnetic bead

Thirty micro liter of magnetic bead suspension were added to lysis buffer (760 µl) and sample (isolate/serum) (140 µl), further the tubes were incubated at room temperature for 8 min. Subsequently, proteinase K (25 µl) was added, to the tubes and further incubated for additional 2 min. The magnetic bead particles were separated and supernatant was discarded. Subsequently, washing buffer (500 µl) containing proteinase K solution (2.5 ml) (20 mg/ml) was added, and the beads were mixed vigorously. Further the mix was incubated for 10 min with washing buffer, the magnetic beads particles were separated by applying magnetic field and the remaining supernatant was pipetted out. The washing step was repeated again following removal of washing buffer and elution buffer (50 µl) was applied to the bead and mixed for uniform suspension. This was further kept at room temperature for 10 min. Finally the beads were separated and eluate was collected in RNase-free/tube by applying magnetic field. The purity and concentration of eluted RNA was carried out in NanoDrop Spectrophotometer (Thermo Fisher Scientific, USA). The suitability of extracted viral RNA for molecular assay was confirmed through Chikungunya specific SYBR green one step qRT-PCR^21^. The eluted RNA was stored at − 80 °C.

### Extraction of viral RNA employing Qiagen kit

The extraction of viral RNA was carried out from infected material using the Qiagen viral RNA mini kit (Qiagen, Germany), according to the manufacturer’s protocol. In brief AVL buffer (560 µl) was added to sample (isolate/serum) (140 µl ) and thoroughly vortexed (15 s), than the mixture was further kept at room temperature (10 min). Subsequently, ethanol (560 µl) was applied to this mixture and this to was passed with the QIAamp spin column. by centrifugation (6000 rpm, 1 min), the spin column residuals were discarded. Rest of this solution was again passed through above mentioned column by recentrifugation as mentioned above. Spin column were further washed using wash buffer AW1 and AW2 (500 µl). Finally viral RNA was extracted in elution buffer (50 µl) added to the centre of the column followed by centrifugation at 8,000 rpm for 2 min. The purity and concentration of viral RNA was analysed with NanoDrop Spectrophotometer (Thermo Fisher Scientific, USA). The suitability of extracted viral RNA for molecular assay was confirmed through Chikungunya specific SYBR green one step qRT-PCR^21^. Finally the eluted RNA (70 µl) was stored at − 80 °C.

### SYBR green real time reverse transcription-PCR

SYBR Green one-step real time qRT-PCR was standardized using the previous reported protocol^21^. Briefly, test samples were assayed in a 25 µl reaction mixtures containing 2X SYBR reaction mix, forward and reverse primers, Reverse transcriptase/Taq mix and template RNA and final volume was adjusted with nuclease free water. No template control (NTC) and positive controls (PTC) were also included during each run. The thermal profile comprises of reverse transcription at 50 °C for 30 min, initial denaturation at 95 °C for 10 min, and 40 cycles of denaturation, primer annealing and extension at 95 °C 30 s, 57 °C 1 min, 72 °C 30 s respectively). The authenticity of the amplified product was determined through analysis of a melting curve after amplification (Tm) with the software of the ABI 7,500 Dx (ABI, USA) according as per the manufacturer’s instructions. Briefly, the temperature was decreased to 57 °C followed by incremental increase in temperature up to 95 °C at a rate of 1 °C/30 s/cycle with continuous measurement of fluorescence.

### Reverse transcription polymerase spiral reaction (RT-PSR)

#### Primer design

The potential target region for designing oligonucleotide primers for RT-PSR amplification were identified with high conservity across the genome with global circulating strains pertaining to all different Chikungunya virus genotypes using ClustalW programme, available in Lasergene 5 package (DNAStar, USA). Also the multiple sequence analysis were also carried out with other closely related alphaviruses i.e. Ross river virus, O’nyong-nyong virus, Semliki Forest virus, Sindbis virus was also carried out to study cross reactivity. Further an outer linker sequence from a botanic gene was appended at 5′ end in both upstream and down stream primer, as required for the polymerase spiral reaction^9^. The details of primer sequence and genomic position is given in Table 3.

**Table 3:** Details of Polymerase spiral reaction primer for detection of Chikungunya virus.

Primer	Sequence (5′-3′)	Genomic position
CHIKV-PSR_FP	acgattcgtacatagaagtatag ACGCAATTGAGCGAAGCAC	10,294–10,312
CHIKV-PSR_RP	gatatgaagatacatgcttagca CTGAAGACATTGGCCCCAC	10,498–10,480

Sequence in upper case refers to target in CHIKV genome (E1 gene) and lower case refers to an exogenous sequence of botanic gene.

#### Optimization of RT-PSR assay

Initial standardization of RT-PSR assay was performed with viral RNA from standard CHIKV culture^9^. The reaction was performed in 25 µl reaction volume which includes Bst DNA polymerase (8U ) (New England Biolabs, US), MMLV RT (1 U) (Promega, US), 40 μM each of both forward (CHIKV-PSR FP) and reverse primers (CHIKV- PSR RP), 2.5 µl 10X ThermolPol reaction buffer (New England Biolabs, US) is comprised off Tris–HCl (20 mM), KCl (10 mM), (NH_4_)_2_SO_4_ (10 mM), MgSO_4_ (2 mM), 0.1% Tween 20), Betaine (0.8 M) (Sigma, US), MgSO_4_ (6 mM), each deoxynucleoside triphosphate (1.4 mM) and template RNA (5 µl). The assay was best optimized at 65 °C for 60 min. Positive template control (PTC) and no template controls (NTC) were included in each run to rule out cross contamination. The reaction was performed using a digital heat block set at 65 °C. Following amplification reaction, 0.2 µl (10,000X conc) SYBR green nucleic acid dye (Lonza, USA) was added to amplified DNA for visual detection in both naked eye or using UV transilluminator. In addition, the real time amplification of the reactions were also recorded in LoopAmp Real time turbidimeter (LA-500, Eiken, Japan). The amplified RT-PSR products were subsequently resolved in 2.5% agarose gel and recorded in gel documentation system (Bio-Rad, USA). RT-PSR experiments were repeated thrice for reproducibility.

### Lyophilization of RT-PSR reagents

D-trehalose was added in the reaction with a concentration of 50% in optimized RT-PSR master mix reagent. The master mix was aliquoted in 20 µl of reaction volume to RT-PSR reaction strips and frozen at − 80 °C for 2 h. Following freezing, the tubes were immediately placed under 0.001 mBar vaccum at − 40 °C for 3 h for lyophilisation in freeze dryer system (Labconco, USA). Freeze dried reaction mix was stored at both room temperature and 4 °C in desiccant containing sealed pouches. The stability of the reagents was studied at an interval of 1 week for 1 month and subsequently at 1 month interval up to 6 months.

### Sensitivity and specificity studies of reverse transcription-PSR assay

The CHIKV 205 bp amplicon pertaining to the target within envelope gene (corresponding to genomic position 10,294–10,498), the same was cloned in pGEM-T easy vetor (Promega, USA) for determination of both sensitivity and specificity. This cloned target within plasmid was linearized by using restriction enzyme Pst I. After RE digestion, linearized fragment were in vitro transcribed with T7 in vitro transcription kit (MBI Fermantas, USA). After in vitro transcription the RNA was purified finally by ethanol precipitation and was further resuspended in nuclease free water. The quantity of CHIKV-RNA transcripts was determined using spectrophotometer and further the RNA molecular copies were calculated.

The detection limit of the Reverse Transcription-PSR assay was determined using tenfold serially diluted in vitro transcribed RNA, which was further added to RT-PSR assay along with positive and no template controls. The appearance of amplification curve and green fluorescence was observed through both real time amplification as well the naked eye visualization. The sensitivity of RT-PSR assay was then determined in both liquid and lyophilized formats. Similarly the same dilutions were also subjected to SYBR Green one-step real-time RT-PCR and the comparative results were observed. The specificity of this assay was determined by including other closely related alphaviruses (O’nyong-nyong virus, Semliki Forest virus, Ross River virus, and Sindbis Virus) and flaviviruses (Dengue virus serotypes 1–4, Japanese encephalitis virus, and West Nile virus). RNA extracted from alpha- and flavivirus infected culture supernatant (10^6,7^ RNA copies/rxn) were used as template in RT-PSR. The same templates were also confirmed for presence of respective genomic targets using alphavirus genus specific end point RT-PCR^28^, and respective flavivirus specific Real time RT-PCR^29–31^.

### Evaluation of RT-PSR with clinical samples

The lyophilized RT-PSR in combination with magnetic bead sample extraction protocol was compared with SYBR Green qRT-PCR in combination with Qiagen RNA extraction protocol. A total of 50 Chikungunya positive and 20 apparently healthy human serum samples were utilized for the evaluation of RT-PSR assay. The panel was first screened by Qiagen RNA extraction and SYBR Green one step Real time RT-PCR and the result was compared with RT-PSR assay. Ten of the randomly selected positive samples were sequenced by double pass Sanger’s sequencing.

## Supplementary information

Supplementary information
