# Supplementary material for: Development of magnetic bead based sample extraction coupled polymerase spiral reaction for rapid on-site detection of Chikungunya virus
Source: Sci Rep. 2020 Jul 15;10:11651. doi: 10.1038/s41598-020-68469-2 (PMC7363856; doi:10.1038/s41598-020-68469-2)

SUPPLEMENTARY INFORMATION for

Development of magnetic bead based sample extraction coupled polymerase spiral reaction for rapid on-site detection of Chikungunya virus

Shashi Sharma[1], Deepak Pardasani[2], Paban Kumar Dash[1*], Manmohan Parida[1], Devendra Kumar Dubey[2]

Virology Division[1], Vertox Division[2], Defence Research Development Establishment, Jhansi Road, Gwalior – 474002, India

Supplementary Files:

Supplementary Table 1: Details of Chikungunya virus positive samples evaluated with Qiagen RNA extraction coupled SYBR green RT-PCR and Magnetic bead RNA extraction coupled lyophilized RT-PSR. The viral RNA copies/rxn was calculated from standard curve drawn using Ct value Vs RNA copies through a serial 10 fold dilution of in vitro transcribed RNA.

Supplementary Fig S1: Basic setup and procedures of magnetic bead based nucleic acid extraction protocol. The protocol includes lysis, washing and elution steps in presence of magnetic field.

Supplementary Fig S2: RT-PSR reaction in liquid format.

A. Reaction set in Dry Bath set at 65^0C
B. Reaction set in Real-time turbidimeter
C. Visual detection of positive and negative RT-PSR reaction through naked eye under normal light
D. Visual detection of positive and negative RT-PSR reaction through naked eye under UV light
E. Amplification curve showing positive reaction in Real-time turbidimeter
F. Agarose gel electrophoresis of RT-PSR amplified product

Supplementary Fig S3: RT-PSR reaction in lyophilized format.

A. Lyophilized RT-PSR reagents
B. Visual detection of lyophilized positive and negative RT-PSR reaction through naked eye under normal light
C. Visual detection of lyophilized positive and negative RT-PSR reaction through naked eye under UV light
D. Lyophilized reagents showing amplification curve of positive reaction in real time turbidimeter
E. Agarose gel electrophoresis of lyophilized RT-PSR amplicons

F. Visual detection of lyophilized RT-PSR reaction in control and samples.

Supplementary Fig S4: Stability of lyophilized RT-PSR reagents.

A. Preparation lyophilized reagents.

B. Packaging and storage of lyophilized reagents at 4 ºC and room temperature.

C. Stability of RT-PSR reagents at 4ºC, at 2 months (C1), 4 months (C2), 6 months (C3) and at room temperature at 1 week (C4).

Supplementary Fig S5: Analysis of cross reactivity of lyophillized RT-PSR using a RNA panel of alphaviruses and flaviviruses. Lane M: 200bp DNA ladder, Lane 1: Chikungunya virus, Lane 2: O'nyong-nyong virus, Lane 3: Semliki Forest virus, Lane 4: Ross River virus; Lane 5: Sindbis virus; Lane 6: Dengue virus type-1 (DENV-1), Lane 7: Dengue virus type-2 (DENV-2), Lane 8: Dengue virus type-3 (DENV-3), Lane 9: Dengue virus type-4 (DENV-4), Lane 10: Japanese Encephalitis virus, Lane 11: West Nile virus, Lane 12: Negative Control

Supplementary Fig S6: Alphavirus genus specific end point RT-PCR showing amplification of 434 bp amplicon. Lane M: 200bp DNA ladder; Lane 1: O'nyong-nyong virus; Lane 2: Semliki Forest virus; Lane 3: Ross River virus; Lane 4: Sindbis Virus; Lane 5: Chikungunya virus; Lane 6: Negative control

Supplementary Fig S7: Taqman qRT-PCR (WNV, DENV 1-4) and SYBR Green qRT-PCR (JEV) showing amplification of Flaviviruses with their virus specific primers. A. West Nile Virus (WNV); B. Japanese Encephalitis Virus (JEV); C. Dengue virus type- 1 (DENV-1); D. Dengue virus type 2 (DENV-2); E. Dengue virus type 3 (DENV-3), F. Dengue virus type 4 (DENV-4).

Supplementary Table 1: Details of Chikungunya virus positive samples evaluated with Qiagen RNA extraction coupled SYBR green RT-PCR and Magnetic bead RNA extraction coupled lyophilized RT-PSR. The viral RNA copies/rxn was calculated from standard curve drawn using Ct value Vs RNA copies through a serial 10 fold dilution of *in vitro* transcribed RNA.

Sample ID	SYBR real time PCR (RNA copies/Rxn)	RT-PSR (Visual detection)
1	$1.32x10^{6}$	+Ve
2	$2.4x\ 10^{3}$	+Ve
3	$1.51x10^{5}$	+Ve
4	$1.51x10^{5}$	+Ve
5	$1.51x10^{5}$	+Ve
6	$1.32x10^{6}$	+Ve
7	$2.4x10^{3}$	+Ve
8	$1.51x10^{5}$	+Ve
9	$1.51x10^{5}$	+Ve
10	$1.32x10^{6}$	+Ve
11	$2.2x\ 10^{5}$	+Ve
12	$1.6x10^{5}$	+Ve
13	$1.8X10^{3}$	+Ve
14	$1.34X10^{6}$	+Ve
15	$1.43X10^{5}$	+Ve
16	$4.8X\ 10^{6}$	+Ve

17	$3.2X\ 10^{4}$	+Ve
18	$1.8X\ 10^{4}$	+Ve
19	$5.6X10^{6}$	+Ve
20	$2.4X\ 10^{3}$	+Ve
21	$1.67X10^{6}$	+Ve
22	$5.8X10^{4}$	+Ve
23	$1.2X10^{3}$	+Ve
24	$2.8X\ 10^{1}$	-Ve
25	$4.6X10^{4}$	+Ve
26	$3.2X10^{4}$	+Ve
27	$8.6X10^{3}$	+Ve
28	$6.7X10^{6}$	+Ve
29	$1.62X10^{4}$	+Ve
30	$2.5X10^{1}$	-Ve
31	$1.82X\ 10^{4}$	+Ve
32	$6.9X10^{2}$	+Ve
33	$2.5X10^{1}$	-Ve
34	$5.9X10^{4}$	+Ve
35	$2.6X10^{3}$	+Ve
36	$6.4X10^{4}$	+Ve
37	$2.8X10^{1}$	-Ve
38	$5.7X10^{4}$	+Ve
39	$4.4X10^{4}$	+Ve

40	$2.9X10^{3}$	+Ve
41	$3.68X10^{4}$	+Ve
42	$1.67X10^{3}$	+Ve
43	$5.4X10^{2}$	+Ve
44	$8.97X10^{2}$	+Ve
45	$3.68X10^{2}$	+Ve
46	$2.87X10^{6}$	+Ve
47	$6.89X10^{2}$	+Ve
48	$5.8X10^{2}$	+Ve
49	$4.89X\ 10^{2}$	+Ve
50	$6.56X10^{6}$	+Ve

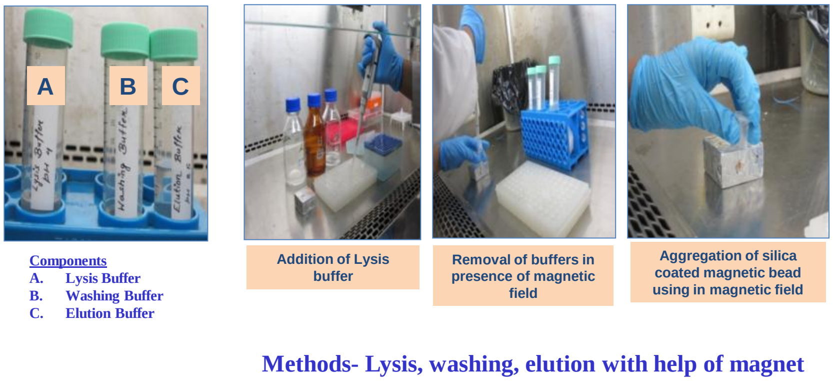

Fig S1: Basic set up and procedures of magnetic bead based nucleic acid extraction method

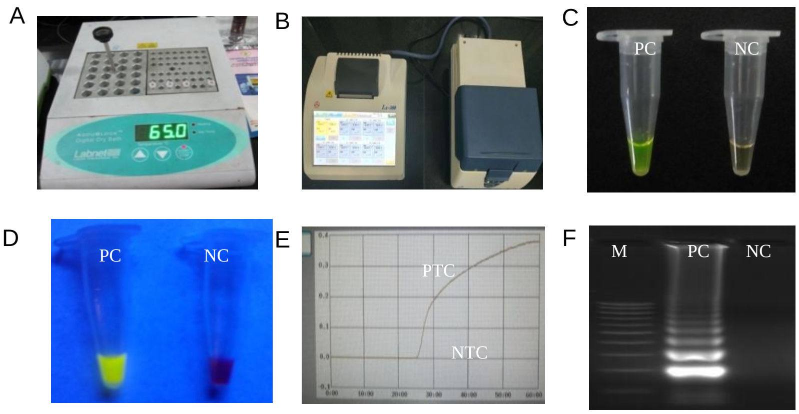

Fig S2: RT-PSR reaction in liquid format.

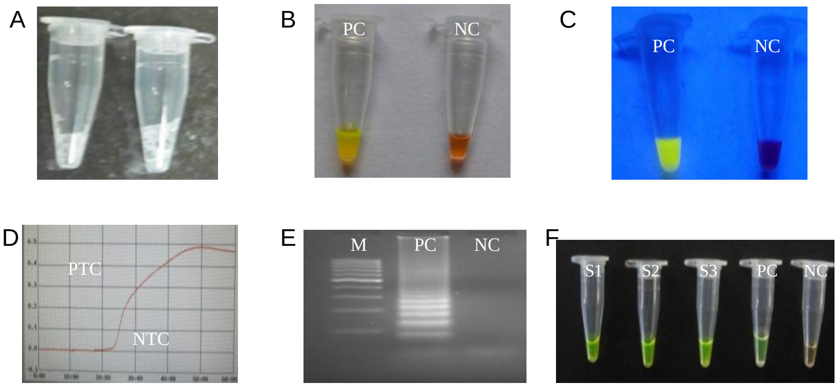

Fig S3: RT-PSR reaction in lyophilized format

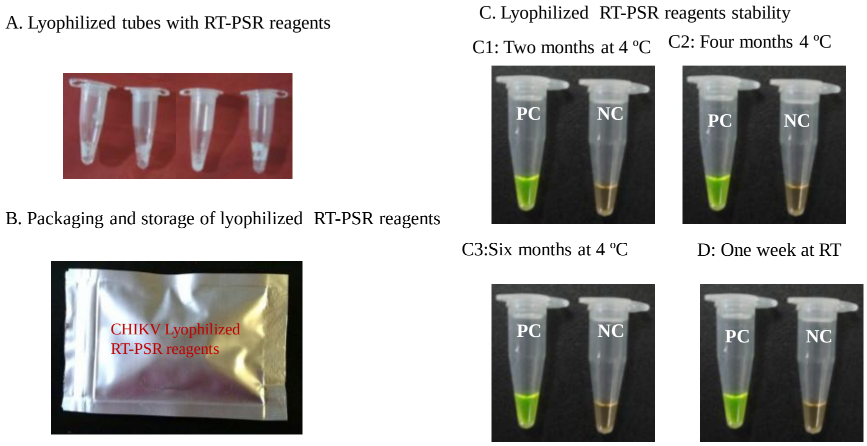

Fig S4: Stability of lyophilized RT-PSR reagents.

Fig S5: Cross reactivity studies with Alpha and flaviviruses

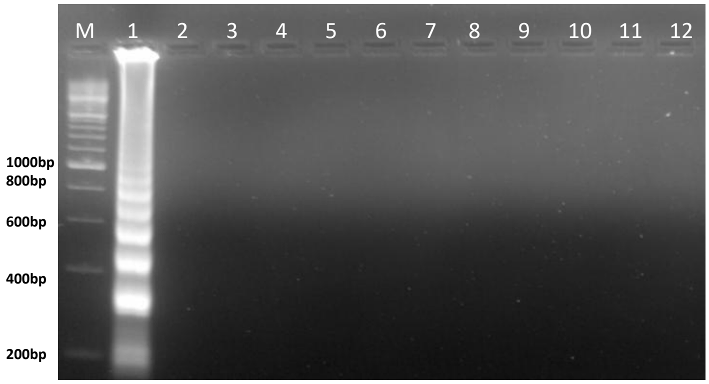

Fig S6: Alphavirus genus specific endpoint RT-PCR

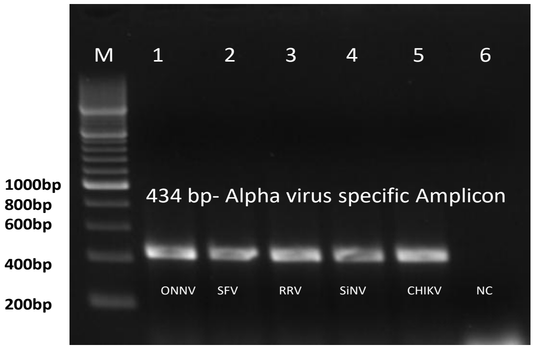

Fig S7 : Respective Flavivirus specific Real-time RT-PCR

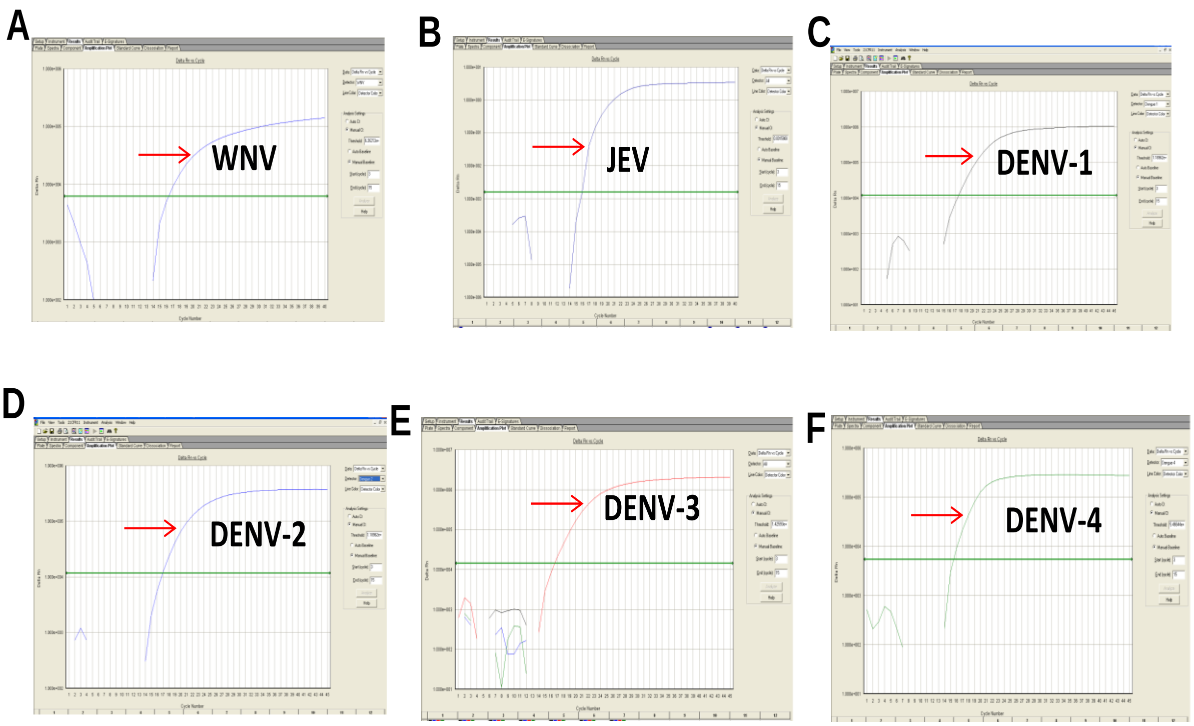

Supplement: Supplementary file 1 — Supplementary information [file 41598_2020_68469_MOESM1_ESM.pdf]
